# Supplementary material for: Prognostic value of mean platelet volume/platelet count ratio in patients with resectable esophageal squamous cell carcinoma: a retrospective study
Source: PeerJ. 2019 Jul 8;7:e7246. doi: 10.7717/peerj.7246 (PMC6622162; doi:10.7717/peerj.7246)
Supplement: Supplemental Information 2 [file peerj-07-7246-s002.docx]

**Codebook**

1. survival status: patients alive or death (0=alive; 1=death)

2. vessel invasion: patients whether have vessel invasion or not (0=No; 1=Yes)

3. adjuvant therapy: patients whether have adjuvant therapy or not (No=0; Yes=1)

4. gender (female=0; male=1)

5. tumor length stage: patients were divided into two groups according to the tumor length with the cut-off value of 3.0 cm (≤3.0=0; >3.0=1)

6. age stage: patients were divided into two groups according to the age with the cut-off value of 60 years (≤60=0; >60=1)

7. tumor location (upper=1; middle=2; lower=3)

8. differentiation (well=1; moderate=2; poor=3)

9. N stage (N0=1; N1=2; N2=3; N3=4)

10. T stage (T1=1; T2=2; T3=3; T4=4)

11. MPV stage: patients were divided into two groups according to the MPV with the cut-off value of 8.5 fl (≤8.5 fl=0; >8.5 fl=1)

12. PC stage: patients were divided into two groups according to the PC with the cut-off value of 200 giga/l (≤200 giga/l=0; >200 giga/l=1)

13. MPV/PC stage: patients were divided into two groups according to the MPV/PC with the cut-off value of 0.04 (≤0.04=1; >0.04=0)

14. CRP stage: patients were divided into two groups according to the CRP with the cut-off value of 10.0 mg/l (≤10.0 mg/l=0; >10.0 mg/l=1)
